# Whole body-electromyostimulation effects on serum biomarkers, physical performances and fatigue in Parkinson’s patients: A randomized controlled trial

**DOI:** 10.3389/fnagi.2023.1086487

**Published:** 2023-02-09

**Authors:** Alessandra di Cagno, Andrea Buonsenso, Marco Centorbi, Luigi Manni, Alfonso Di Costanzo, Giusy Casazza, Attilio Parisi, Germano Guerra, Giuseppe Calcagno, Enzo Iuliano, Marzia Soligo, Giovanni Fiorilli, for The WB-EMS Parkinson’s Group, Francesco Lena

**Affiliations:** Department of Medicine and Health Sciences, University of Molise, Campobasso, Italy; Neuromed Institute IRCCS, IS, Pozzilli, Italy; Department of Clinical and Experimental Medicine, University of Catanzaro “Magna Græcia”, Italy; ^1^Department of Movement, Human and Health Sciences, University of Rome “Foro Italico”, Rome, Italy; ^2^Department of Medicine and Health Sciences, University of Molise, Campobasso, Italy; ^3^Institute of Translational Pharmacology and Cellular Biology and Neurobiology Institute (CNR), National Research Council (CNR), Rome, Italy; ^4^Centre for Research and Training in Medicine of Aging, Department of Medicine and Health Sciences, University of Molise, Campobasso, Italy; ^5^Faculty of Psychology, eCampus University, Novedrate, Italy

**Keywords:** Parkinson’s disease, neurotrophic factors, physical activity, functional capacity, muscle stimulation

## Abstract

**Background:**

Whole-body electromyostimulation (WB-EMS) was never previously applied to Parkinson’s disease (PD) patients. This randomized controlled study aimed to find the most effective and safe WB-EMS training protocol for this population.

**Methods:**

Twenty-four subjects (age: 72.13 ± 6.20 years), were randomly assigned to three groups: a high-frequency WB-EMS strength training group (HFG) (rectangular stimulation at 85 Hz, 350 μs, 4 s stimulation/4 s rest), a low-frequency WB-EMS aerobic training group (LFG) (rectangular stimulation 7 Hz, 350 μs, with a continuous pulse duration), and an inactive control group (CG). Participants of the two experimental groups underwent 24 controlled WB-EMS training sessions, with a duration of 20 min each, during 12-week intervention. Serum growth factors (BDNF, FGF-21, NGF and proNGF), α-synuclein, physical performance and Parkinson’s Disease Fatigue Scale (PFS-16) responses were analyzed to evaluate the pre-post variation and differences among groups.

**Results:**

Significant interactions of Time*Groups were detected for BDNF (Time*Groups *p* = 0.024; Time*CG, *b* = −628, IC95% = −1,082/−174, *p* = 0.008), FGF-21 (Time*Groups *p* = 0.009; Time*LFG *b* = 1,346, IC95% = 423/2268, *p* = 0.005), and α-synuclein (Time*Groups *p* = 0.019; Time*LFG *b* = −1,572, IC95% = −2,952/−192, *p* = 0.026). *Post hoc* analyses and comparisons of ΔS (post–pre), performed independently for each group, showed that LFG increased serum BDNF levels (+ 203 pg/ml) and decreased α-synuclein levels (−1,703 pg/ml), while HFG showed the opposite effects (BDNF: −500 pg/ml; α-synuclein: + 1,413 pg/ml). CG showed a significant BDNF reduction over time. Both LFG and HFG showed significant improvements in several physical performance outcomes and the LFG showed better results than HFG. Concerning PFS-16, significant differences over time (*b* = −0.4, IC95% = −0.8/−0.0, *p* = 0.046) and among groups (among all groups *p* < 0.001) were found, and the LFG exhibited better results than the HFG (*b* = −1.0, IC95% = −1.3/−0.7, *p* < 0.001), and CG (*b* = −1.7, IC95% = −2.0/−1.4, *p* < 0.001) with this last one that worsened over time.

**Conclusion:**

LFG training was the best choice for improving or maintaining physical performance, fatigue perception and variation in serum biomarkers.

**Clinical trial registration:**

https://www.clinicaltrials.gov/ct2/show/NCT04878679, identifier NCT04878679.

## 1. Introduction

The technology based on whole-body electromyostimulation (WB-EMS) is a time-efficient, non-invasive, and undemanding training modality involving the simultaneous stimulation of large muscle areas with dedicated individual intensity per muscle group while performing static or dynamic voluntary movements (Kemmler et al., 2018). Electromyostimulation, which alters physiological recruitment patterns, favors the activation of fast motor units in addition to slow ones and offers advantages, particularly for elderly people (Maffiuletti, 2010; de Oliveira et al., 2021); it has been shown that this methodology leads to significant improvements on sarcopenia, muscle mass and strength parameters (de Oliveira et al., 2022). Therefore, combining electromyostimulation and functional movements allows a high intensity of active work with limited effort (Pano-Rodriguez et al., 2019).

Parkinson’s Disease (PD) patients cannot or are unwilling to adhere to conventional exercise programs because of physical and/or mental limitations, while the time-efficiency and highly individualized setting of WB-EMS training make this methodology particularly suitable for PD patients. In our previous study, we acutely applied WB-EMS training to PD patients, revealing immediate improvements even after only one administration (Fiorilli et al., 2021).

The neuropathological hallmark of PD is the selective degeneration of dopaminergic neurons, representing the last step of a complex degenerative cascade of events underlying neuronal cell death. Aggregated α-synuclein deposition in Lewy bodies seems to play a causative role in mitochondrial damage onset, compromising striatal synaptic functions and inducing oxidative stress, playing an undoubted role in neuronal cell death (Bellucci et al., 2020; Fleming et al., 2022). The α-synuclein has gained attention as a surrogate biomarker for PD, because abnormal accumulation of this protein in cerebrospinal fluid, blood plasma and saliva may reflect the abnormalities found in the brains of PD patients. A recent meta-analysis confirmed that total plasma α-synuclein levels are higher in PD patients than in healthy control (Bougea et al., 2019).

In PD patients, fibroblast growth factor (FGF) is strongly associated with neuroinflammation, reducing the misfolding of α-synuclein and improving the survival rate of dopaminergic neurons (Kaminska et al., 2022).

An insufficient neuronal supply of brain-derived neurotrophic factors (BDNF) has been shown to cause a deficit in synaptic plasticity in PD patients (Allen et al., 2013). Several lines of evidence indicate that physical exercise increases growth factors release and synaptic connectivity and alleviates the loss of dopaminergic neurons, resulting in improved motor deficits observed in PD (Lauzé et al., 2016). Muscle tension and rigidity, slow movements, cognitive impairments and fatigue clinically characterize PD (Vermeiren et al., 2020). Pharmacological intervention is less effective on PD fatigue (Azevedo et al., 2022).

Although pharmacological therapeutic approaches mitigate PD symptoms, long-term administration generates serious side effects, such as rhabdomyolysis (Turcano et al., 2018). Conversely, regular exercise affords a neuroprotective effect against PD, enhancing the release of growth factors (Schaeffer et al., 2022) along with multiple related beneficial effects in enhancing strength, balance and promoting quality of life (Tang et al., 2019; Fiorilli et al., 2021), attenuating PD symptom progression (Johansson et al., 2022). Therefore, we designed two exercise protocols combined with WB-EMS, used as additional treatment, during a 12-week training period lasting only 20 min, two times per week.

This study aimed to examine the effectiveness of WB-EMS superimposed on different exercise protocols based on strength or aerobic training, on growth factors, α-synuclein serum levels and physical performance, in PD patients, compared with inactivity.

## 2. Material and methods

### 2.1. Study design

This Randomized Controlled Trial (RCT) study was designed as a three-arm parallel single-center study with two experimental groups and a control group (CG). The study is fully registered under www.clinicaltrials.gov (NCT04878679).

### 2.2. Participants

Twenty-four participants (age: 72.13 ± 6.20 years) were recruited and randomly assigned to the high frequency-WB-EMS strength training group (HFG), low frequency-WB-EMS aerobic training group (LFG) and CG (Table 1). All the participants underwent a specialist medical examination and maintained their pharmacological treatment (levodopa). The following inclusion criteria were applied for enrolment: age from 50 to 80 years; clinical diagnosis of PD in the stage from 1 (mild) to 3 (moderate) assessed by the Hoehn and Yahr scale (Hoehn and Yahr, 1967); no simultaneous participation in any type of supervised physical activity; Sedentary lifestyle. The following exclusion criteria were instead applied: mini-mental state examination (MMSE) score of less than 24; inability to walk for 6 min without assistance; the presence of a medical condition influencing the cognitive and/or motor functions; presence of any counter indication for the utilization of EMS, such as cardiovascular diseases, stents, cardiac pacemakers, and diabetes mellitus, verified by medical certification. The study was conducted at University of Molise and was approved by the Local Bioethical Committee (11487/2020). All the participants provided written informed consent.

**TABLE 1:** Characteristics of the sample.

	Low-frequency group (LFG)	High-frequency group (HFG)	Control group (CG)
Number of participants and gender	8 participants 5 males 3 females	8 participants 7 males 1 female	8 participants 6 males 2 females
	**Means ± SD**	**Means ± SD**	**Means ± SD**
Age (years)	73.13 ± 2.85	72.37 ± 7.40	70.87 ± 7.77
Hoehn and Yahr scale	1.44 ± 0.62	1.87 ± 0.35	2.19 ± 0.65
MMSE score	26.49 ± 1.06	26.64 ± 2.63	26.57 ± 2.58

MMSE, mini-mental state examination.

### 2.3. Blinding

The researchers involved in the general conditions and physical performances’ assessment, as well as the researchers involved in the data analysis were blinded to randomization assignment. The participants of the study were blinded concerning the training program performed by other participants and each participant was individually trained and tested. Lastly, the trainers who applied the intervention were blinded concerning the training program performed by other groups. During each assessment, participants and researchers could not provide or request information about the exercise program that participants were performing.

#### 2.3.1. Sample size computation

Sample size was calculated based on BDNF data (considered the study’s primary outcome) that was reported in a similar study, using G*Power (version 3.1.9.6; written by Franz Faul, University of Kiel, Germany). The following design specifications were considered: test family = *F* tests; statistical test = analysis of variance (ANOVA) repeated measures, between factors; α = 0.05; (1–β) = 0.95; effect size *f* = 0.8; number of groups = 3; number of measurements = 2. Sample size estimation indicated 24 participants (eight per group) with a critical *F* value of 3.467 (Siqueira et al., 2018).

#### 2.3.2. Enrollment process

The participants were enrolled by the staff of Center of Research in Medicine of Aging of Molise, not involved in the study, in September 2021. The procedures of the study were conducted from October 2021 to December 2021.

#### 2.3.3. Randomization

Eligible participants were randomized into three groups, HFG, LFG, and CG, using 1:1:1 blind randomization. A computer-generated random allocation sequence was used by an independent statistician who assigned participants to three groups. The list was kept in a numbered envelope containing the generated allocation.

### 2.4. Participants’ involvement

The participants were enrolled by the staff of Center of Research in Medicine of Aging of Molise, not involved in the study. Patients, neurologists, researchers, and trainers were involved in a trial committee. During several community meetings, the trial team improved its understanding of research purposes and processes. This involvement established links with the patients’ community, acting as a significant bridge between researchers and patients to support, monitor and adapt the intervention conditions. Patients or the public were not involved in the design, or conduct, or reporting, or dissemination plans of our research.

### 2.5. Experimental procedures

Each subject randomly assigned to the two experimental groups [high frequency-WB-EMS strength training group (HFG), low frequency-WB-EMS aerobic training group (LFG) underwent 24 controlled WB-EMS training sessions during 12 week-intervention, advised and accompanied by a trained and licensed WB-EMS trainer]. A familiarization session based on a curtailed training session with moderate stimulus intensity was performed before the first intervention. The intensity of the stimuli during this familiarization session was set at 5 out of a maximum of 10 according with the Borg’s CR10 rate of perceived exertion scale (RPE) (Hutchinson et al., 2018). This session aimed to learn movement patterns (i.e., proper techniques of the exercises) and to adapt participants to the electric stimuli. Each muscle group was strained in intervals to determine each participant’s subjective maximum, defined by the point at which each person gave the signal to stop the strain, as the maximum degree of strain that may be easily tolerated. The last output of the intensity stimulation value by the WB-EMS was recorded. Five minute-impulse familiarization was also performed before each training session.

The CE-certified medical EMS equipment Miha Bodytec II (Miha Bodytec, Gersthofen, Germany) was used according to the manufacturer instructions. The system consists of a control station with an integrated display and control options for individual muscle groups. Participants were equipped with functional underwear, recommended by the manufacturer, on which a vest (upper body) and belts (arms, legs, and gluteal muscles) were individually tightly adjusted before each training session.

The HFG underwent 20 min of strength training combined with WB-EMS, using the recommended protocol (rectangular stimulation at 85 Hz, 350 μs, 4 s stimulation/4 s rest) (Kemmler et al., 2018). The training consisted of the following five active strength exercises: half squat, full squat, bent over, core rotation, and crunch. Participants were required to perform 4 s of maximal isometric contraction alternated to a 4 s of static rest and were encouraged to perform with maximal effort during the WB-EMS impulse. Every 3 weeks of training, the trainer applied a higher external resistance thought dumbbells (from 0.5 to 3 kg). The load progression, in terms of external resistance, was pre-programmed over time, instead, the intensity of the electrical stimulus was established for each participant and for each muscle group at the beginning of each training session, to be tolerable for them according to the RPE. In other words, as the training state improved, the intensity of stimulation for each muscle district was also set at higher levels, corresponding to their RPE and based on their current tolerability.

The LFG underwent 20 min of aerobic training, performed on a rowing machine, combined with WB-EMS, using the recommended protocol (rectangular stimulation 7 Hz, 350 μs, with a continuous pulse duration) (Kemmler et al., 2010). The intensity progression of the training was based on the reserve heart rate (HRR) starting from 60% HRR in the 1st week and increasing the intensity of 5% every 3 weeks, up to 80% HRR in the 12th week. Maximum heart rate was calculated using Tanaka’s formula (Tanaka, 2001).

The control group (CG) did not carry out any kind of training and was encouraged to keep the usual daily routines.

### 2.6. Outcome measures

Blood samples were collected before and after the experimental procedures to assess growth factors (FGF-21, BDNF, proNGF, and NGF) and α-synuclein serum levels. Blood samples were centrifuged at 3,000 × *g* for 10 min and the serum fractions were collected. ELISA analysis for FGF-21 (Human Duoset ELISA DY2539, R&D system), BDNF (Human Duoset ELISA DY248, R&D system), α-synuclein (Human Duoset ELISA DY1338, R&D system) and NGF (Human Duoset ELISA DY256, R&D system) was performed according to manufacturer’s protocol. ELISA analysis for proNGF was perform as described (Soligo et al., 2015). Before and after the experimental procedures, the Senior Fitness Test battery (Rikli and Jones, 2013) was administered to assess the physical performance level. This test battery consists of the following tests: Sit-to-stand test, for lower limbs’ endurance (Jones et al., 1999); 8 foot up-and-go test, for dynamic balance and agility skills (Cancela et al., 2012); 6-minute walking test, to assess the cardiorespiratory fitness (Enright, 2003); chair sit-and-reach test, for trunk and lower limbs’ flexibility (Jones et al., 1998); Soda-Pop test, for the oculo-manual coordination (Clark, 1989); and Tinetti’s test, for balance, walking and fall-risk evaluation (Tinetti et al., 1986). Finally, the Parkinson Fatigue Scale 16 (PFS-16) (Brown et al., 2005) was used to evaluate fatigue perception and its impact on daily life functions. The scale is composed by 16 items, each of which is assigned a score from 1 (best score) to 5 (worst score). The final score was calculated as an average value of the 16 item scores.

### 2.7. Statistical analyses

SPSS version 26.0 for Windows (SPSS Inc., Chicago, IL, USA) was used for data analyses and *P*-value < 0.05 was considered as statistically significant.

Baseline homogeneity of the examined variables among groups was assessed using the non-parametric Kruskal-Wallis test, while Shapiro-Wilk test was used to evaluate normality.

Statistical analysis and presentation are consistent with the CHAMP statement (Mansournia et al., 2021).

*Serum biomarkers:* A Generalized linear mixed-effect models analysis with repeated measures (GLMM) was used to evaluate significant differences between pre and post-tests and among the three groups (fixed effect Time and Group, respectively). When no difference between groups was detected, the interaction Time*Groups was investigated to evaluate whether the three groups had a different trend over time. Participants were considered as random effect. Five biomarkers (BDNF, FGF-21, proNGF, NGF, and α-synuclein) serum levels were analyzed as target variables. Bonferroni correction for multiple comparisons was applied. Due to the elevate heterogeneity of the analyzed serum biomarkers at baseline, and the presence of significant interaction Time*Groups, a supplementary analysis was performed among the variation from baseline scores (Delta Score, ΔS). ΔS scores comparisons were performed using One-way Kruskal–Wallis H test. The three groups were considered as between factors of the analysis, whereas the five biomarkers ΔS were considered as independent variables. When significant differences among the three groups were found, Wilcoxon Test was used to evaluate variation over time for the three groups analyzed separately.

*Physical performances and PFS-16:* GLMM with repeated measures was used to evaluate significant differences between pre and post-tests and among the three groups. Also in this case, when no difference between groups was detected, the interaction Time*Groups was investigated to evaluate whether the three groups had a different trend over time. Participants were considered as random effect. Physical performance tests’ scores as well as PFS-16 were used as target variables. Bonferroni correction for multiple comparisons was applied.

## 3. Results

**FIGURE 1:**
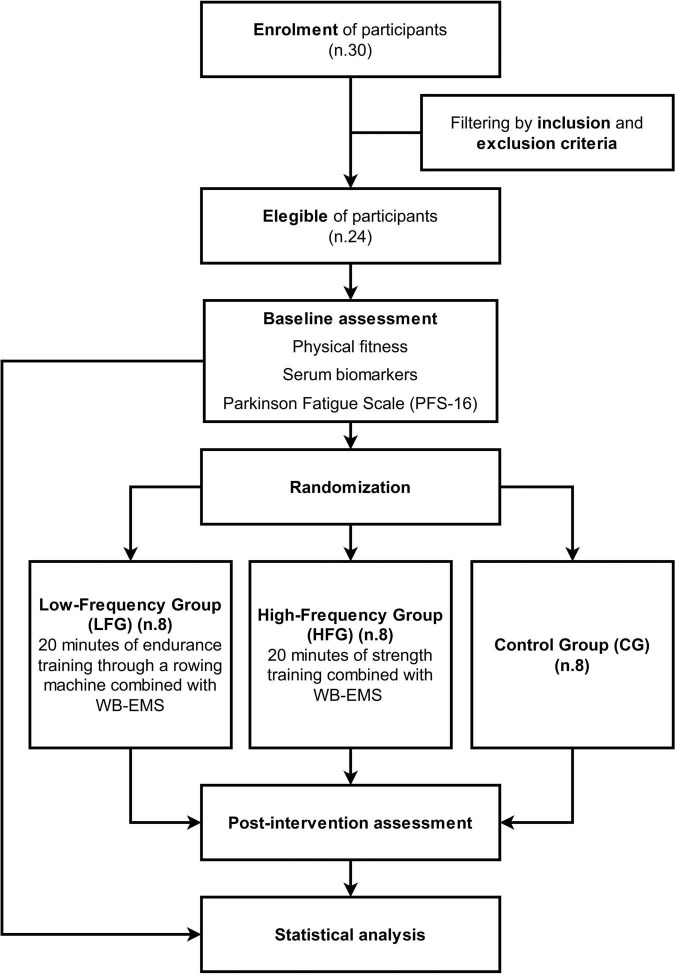
Flowchart of the study.

As previously stated, this study is a RCT designed as a three-arm parallel single-center study with two experimental groups and a control group (Figure 1). After the randomization the three groups resulted homogeneous at baseline relatively to the age, gender, Hoehn and Yahr scale, MMSE score, serum biomarkers, physical fitness test scores and PFS-16 score.

The statistical analysis performed at the end of the procedures showed the results that were reported below.

### 3.1. Results of analysis of serum biomarkers

The GLMM with repeated measures showed significant differences over time in FGF-21 values (*b* = 722, IC95% = 150/1,294, *F*_(1,44)_ = 6.474, *p* = 0.015) with no differences among groups. Significant interactions Time*Groups was detected for α-Synuclein (Time*Groups *F*_(3,44)_ = 3.676, *p* = 0.019; Time*LFG *b* = −1,572, IC95% = −2,952/−192, p = 0.026), BDNF (Time*Groups *F*_(2)_ = 3.481, *p* = 0.024; Time*CG, *b* = −628, IC95% = −1,082/−174, *p* = 0.008), FGF-21 (Time*Groups *F*_(2)_ = 4.323, *p* = 0.009; Time*LFG *b* = 1,346, IC95% = 423/2268, *p* = 0.005). For these reasons a supplementary analysis was performed on ΔS for these three proteins levels.

*A -Synuclein:* The analysis showed a significant difference in α-Synuclein serum levels (*H*_(2)_ = 7.898, *p* = 0.019) among the ΔS of the three groups. The Pairwise comparisons showed a significant difference between LFG and HFG α-Synuclein variation (*p* = 0.016): LFG showed an average decrease of −1,703 pg/ml (in seven of the eight participants); HFG showed an average increase of 1,413 pg/ml (in seven of the eight participants). The CG showed an average decrease of −779 pg/ml (in six of the eight participants). However, the Wilcoxon Signed-Rank Test showed that only the decrease found in LFG was significant over time (pre-score vs. post-score, *p* = 0.046).

*BDNF:* The analysis showed a significant difference in BDNF serum levels (*H*_(2)_ = 6.740, *p* = 0.034) among the ΔS of the three groups. Pairwise comparisons showed a significant difference between LFG and CG (*p* = 0.049): the LFG showed an average BDNF increase of 203 pg/ml (in five of the eight participants), whereas HFG showed an average decrease of −500 pg/ml (in five of the eight participants). CG showed an average BDNF reduction of −564 pg/ml (in overall the eight participants of this group). The Wilcoxon Signed-Rank Test showed that the reduction found CG was significant over time (pre-score vs. post-score, *p* = 0.002).

*FGF-21:* The analysis did not showed significant differences in the FGF-21 serum levels among the ΔS of the three groups (*H*_(2)_ = 5.495, *p* = 0.064). However, the *p*-value resulted close to the significance. For this reason, pairwise comparisons and Wilcoxon signed-rank test were performed. Pairwise comparisons did not show significant differences among groups, however, the LFG showed an average FGF-21 increase of 1,346 pg/ml (five of the eight participants), and the HFG showed an average increase of 944 pg/ml (in overall the eight participants), whereas CG showed an average FGF-21 reduction of −124 pg/ml. Furthermore, the Wilcoxon Signed-Rank Test showed that the FGF-21 variation in HFG was significant over time (pre-score vs. post-score, *p* = 0.012), whereas the LFG and CG variation was not significant over time.

*NGF and ProNGF:* No significant results in GLMM analysis for Time, Groups, or interaction Time*Groups, for these serum biomarkers, were found, therefore no supplementary analyses were performed. The detailed results of the analyses were reported in Table 2.

**TABLE 2:** Results obtained by the neurotrophic factors analysis.

	α-syn pre (pg/ml)	α-syn post (pg/ml)	α-syn Δ (pg/ml)	BDNF pre (pg/ml)	BDNF post (pg/ml)	BDNF Δ (pg/ml)	FGF-21 pre (pg/ml)	FGF-21 post (pg/ml)	FGF-21 Δ (pg/ml)
Median (IQR) of LFG	3012.4 (2756.7)	1713.9 (2390.3) *	−1537.2 (1631.9) ^B^	1991.2 (1356.2)	2042.2 (649.2)	174.9 (557.2) ^A^	844 (238.9)	2411.4 (3378.4)	1248.9 (3282.8)
Median (IQR) of HFG	3453.1 (3262.7)	3742.8 (3755.6)	1318.1 (1025.1) ^C^	2131.5 (718.4)	1999.4 (1229.7)	−548.6 (1028.1)	451.3 (768.3)	1233.9 (1457.8) *	231.4 (1223.9)
Median (IQR) of CG	2327.5 (3207.1)	3264 (1818)	−949.2 (1867.4)	1657.9 (1185.1)	862 (870) *	−524.2 (415.1) ^C^	817 (709.7)	613.6 (578)	19.3 (552.8)
IC 95% lower/upper limit of LFG	2879.2/6962.5	141.7/7623.1	−2870.7/−1090.8	680.7/2705.4	1003.3/2468.3	−72.9/1330.2	534.1/1153.8	428.4/4025.2	−605.6/3182.2
IC 95% lower/upper limit of HFG	1378.3/5785.6	3012.4/9848.2	390.9/2461.8	1713.3/2724	845.5/2354.1	−1093.4/99.1	163.2/1091.8	365/4126.9	68.9/2043.3
IC 95% lower/upper limit of CG	1775.8/5326.9	1378.3/3893.8	−2270.1/1236.6	1064.9/2720.9	627.4/1912.7	−808.2/−249	334.2/1616.1	319/1334.4	−1019.1/260.4
	**NGF pre (pg/ml)**	**NGF post (pg/ml)**	**NGF Δ (pg/ml)**	**ProNGF pre (pg/ml)**	**ProNGF post (pg/ml)**	**ProNGF Δ (pg/ml)**			
Median (IQR) of LFG	16.4 (27.1)	14.9 (18.7)	−2.9 (7.3)	20.3 (214.6)	8.1 (89.7)	−7.5 (41.3)			
Median (IQR) of HFG	37.9 (43.5)	26.4 (64.6)	−7.5 (40.7)	55.7 (132.8)	37.5 (125.8)	−3.8 (29.4)			
Median (IQR) of CG	35 (32.5)	17.8 (23)	−10.1 (40.3)	33.4 (12.4)	47 (170.3)	10.4 (69.3)			
IC 95% lower/upper limit of LFG	0/29.2	0/23.5	−11.5/0.7	2.2/299.2	2.2/326.1	−135.3/0.8			
IC 95% lower/upper limit of HFG	6.4/93.3	6.4/143.9	−37.6/47.6	2.2/249.1	2.2/224.8	−39.6/1.6			
IC 95% lower/upper limit of CG	0/44	9.2/58.2	−34.8/23.2	18.8/44.8	25.6/303	−7/154.6			

LFG, low frequency group; HFG, high frequency group; CG, control Group; IQR, interquartile range; IC 95%, 95% confidence interval for median. α-syn, α-synuclein; BDNF, brain derived neurotrophic factor; FGF-21, fibroblast growth factor 21; NGF, nerve growth factor; ProNGF, nerve growth factor precursor.

* = statistically significant differences compared with Pre-test (*p* < 0.05); ^A^ = statistically significant differences compared with CG (p < 0.05); ^B^ = statistically significant differences compared with HFG (p < 0.05); ^C^ = statistically significant differences compared with LFG (*p* < 0.05).

### 3.2. Results of analysis of physical fitness test and PFS-16

*Arm-Curl test:* The GLMM with repeated measures showed significant differences over time in the Arm-Curl test–dominant arm (*b* = 4.5, IC95% = 2.8/6.2, *F*_(1,44)_ = 30.211, *p* < 0.001) and among groups (*F*_(2,44)_ = 9.764, *p* < 0.001). The pairwise comparisons indicated significant differences among CG *vs.* LFG (*b* = −6.2, IC95% = −9.1/−3.4, *p* < 0.001) and HFG (*b* = −3.9, IC95% = −6.8/−1.0, *p* = 0.009). Significant differences were found in the Arm-Curl test–non-dominant arm, over time (*b* = 4.5, IC95% = 2.7/6.2, *F*_(1,44)_ = 28.515, *p* < 0.001) and among groups (*F*_(2,44)_ = 5.465, *p* = 0.008). The pairwise comparisons showed significant differences among CG vs. LFG (*b* = −3.8, IC95% = −6.3/−1.3, *p* = 0.004) and HFG (*b* = −3.3, IC95% = −5.8/−0.7, *p* = 0.012).

*Sit-to-Stand test:* Other significant differences were found in the Sit-to-Stand test, both over time (*b* = 1.8, IC95% = 0.8/2.8, *F*_(1,44)_ = 13.848, *p* = 0.001) and among groups (*F*_(2,44)_ = 3.600, *p* = 0.036). The pairwise comparisons showed significant differences among CG *vs.* LFG (*b* = −2.8, IC95% = −5.2/−0.5, *p* = 0.019) and HFG (*b* = −2.5, IC95% = −4.9/−0.2, *p* = 0.034).

*8 Foot Up-and-Go test:* Significant differences were found in the 8 Foot Up-and-Go test, both over time (*b* = −1.3, IC95% = −2.3/−0.3, *F*_(1,44)_ = 6.923, *p* = 0.012) and among groups (*F*_(2,44)_ = 5.188, *p* = 0.009). The pairwise comparisons showed significant differences among CG *vs.* LFG (*b* = 2.7, IC95% = 0.7/4.8, *p* = 0.009) and HFG (*b* = 2.8, IC95% = 0.8/4.8, *p* = 0.007).

*Tinetti’s test:* Significant differences were found in the Balance test, both over time (*b* = 1.9, IC95% = 0.4/3.4, *F_(1_,_44)_* = 6.631, *p* = 0.013) and among groups (*F_(2_,_44)_* = 4.145, *p* = 0.022) with the pairwise comparisons indicating significant differences among LFG vs. HFG (*b* = 4.1, IC95% = 1.0/7.2, *p* = 0.011) and GC (*b* = 3.5, IC95% = 0.4/6.7, *p* = 0.027).

*6-Minute Walking test*: Significant differences were found in the 6-Minute Walking test, both over time (*b* = 33.6, IC95% = 0.4/66.8, *F*_(1,44)_ = 4.165, *p* = 0.047) and among groups (*F*_(2,44)_ = 3.466, *p* = 0.040). The pairwise comparisons showed significant differences among LFG vs. HFG (*b* = 62.6, IC95% = 7.6/117.5, *p* = 0.027) and GC (*b* = 61.8, IC95% = 6.8/116.8, *p* = 0.028).

*Handgrip test*, *Soda-Pop test and in Sit-and-Reach test:* No significant differences over time and/or among groups were found in these tests. Similarly, no significance was found in the interactions Time*Groups.

*PFS-16:* Concerning the PFS-16, significant differences were found over time (*b* = −0.4, IC95% = −0.8/0.0, *F*_(1,44)_ = 4.200, *p* = 0.046) and among groups (*F*_(2,44)_ = 79.357, *p* < 0.001). The pairwise comparisons showed significant differences among CG *vs.* LFG (*b* = 1.7, IC95% = 1.4/2; *p* < 0.001) and HGF (*b* = 0.7, IC95% = 0.4/1, *p* < 0.001) but also between LFG *vs*. HFG (*b* = −1, IC95% = −1.3/−0.7, *p* < 0.001).

The results of the tests were reported in Table 3.

**TABLE 3:** Results obtained by the physical fitness test and PFS-16.

	Arm-Curl dominant arm pre (score)	Arm-Curl dominant arm post (score)	Arm-Curl non-dominant arm pre (score)	Arm-Curl non-dominant arm post (score)	Sit-to-Stand pre (score)	Sit-to-Stand post (score)	Soda-Pop pre (seconds)	Soda-Pop post (seconds)
Median (IQR) of LFG	16 (2.75)	23.5 (1.5) * ^A^	16 (3.75)	22 (2.5) *^A^	8.5 (3.5)	13 (3) *^A^	11.93 (2.02)	12.37 (1.63)
Median (IQR) of HFG	14 (2.5)	20.5 (2.5) * ^A^	15 (2.75)	21.5 (3.5) *^A^	8.5 (1.75)	12 (2.75) *^A^	13.97 (9)	15.01 (6.3)
Median (IQR) of CG	17 (8)	16.5 (7.25)^BC^	17 (4)	16 (3.5) ^BC^	10 (0.5)	9 (1) # ^BC^	11.25 (1.99)	11.74 (3.29)
IC 95% lower/upper limit of LFG	13/18	21/24	12/18	20/24	8/13	10/16	9.72/14.1	11.93/14.02
IC 95% lower/upper limit of HFG	12/16	19/23	13/18	20/25	8/12	11/16	10.49/24.29	11.32/20.11
IC 95% lower/upper limit of CG	14/22	13/21	15/19	14/19	9/11	8/9	10.74/12.92	9.97/14.08
	**8 Foot Up-and-Go pre (seconds)**	**8 Foot Up-and-Go post (seconds)**	**Sit-and-Reach pre (cm)**	**Sit-and-Reach post (cm)**	**Handgrip dominant hand pre (kg)**	**Handgrip dominant hand post (kg)**	**Handgrip non-dominant hand pre (kg)**	**Handgrip non-dominant hand post (kg)**
Median (IQR) of LFG	14.02 (3.75)	11.09 (1.9) *^A^	−4.5 (8.75)	0 (7.75)	31.5 (9.1)	30.15 (6.4)	26.45 (12.83)	30.75 (12.1)
Median (IQR) of HFG	13.58 (3.89)	10.45 (2.47) *^A^	−4.5 (7)	−3 (8.25)	30.75 (11.23)	30.7 (10.05)	27.65 (13.13)	24.1 (11.83)
Median (IQR) of CG	13.64 (1.41)	15.27 (2.12) #^BC^	−3 (6.5)	−2.5 (5.75)	30.95 (6.73)	32.9 (6.83)	28.9 (8.9)	27.5 (11.3)
IC 95% lower/upper limit of LFG	11.64/15.6	9.48/12.29	−7/7	−8/2	23.9/39.3	26.4/40.3	21/34.2	21.2/38.4
IC 95% lower/upper limit of HFG	11.26/15.78	8.91/13.36	−7/0	−7/8	20.7/33.5	21.1/35.5	16.8/32.7	17.4/32.9
IC 95% lower/upper limit of CG	12.43/14.34	13.82/16.97	−7/4	−6/2	19.9/33.9	20.2/34	18.9/34.7	16.9/36
	**6-Minute Walking pre (meters)**	**6-Minute Walking post (meters)**	**Balance pre (score)**	**Balance post (score)**	**PFS-16 pre (score)**	**PFS-16 post (score)**		
Median (IQR) of LFG	347 (89.13)	469 (94.25) *^AB^	21.5 (3)	28 (2) *^AB^	3.23 (0.3)	1.8 (0.25) *^AB^		
Median (IQR) of HFG	280.5 (49.3)	267.5 (41.25) ^C^	21.5 (4.5)	21 (4) ^C^	3.13 (0.45)	2.8 (0.1)^AC^		
Median (IQR) of CG	302.5 (46.5)	287.5 (30.25) ^C^	22 (3.25)	22.5 (2.5) ^C^	3 (0.48)	3.8 (0.47) # ^BC^		
IC 95% lower/upper limit of LFG	304/395	430/555	18/24	23/28	3.1/3.4	1.6/2		
IC 95% lower/upper limit of HFG	270.5/334.5	260/320	17/26	17/27	2.8/3.4	2.8/3.2		
IC 95% lower/upper limit of CG	278/350	270/322	20/24	19/23	2.5/3.5	3.31/4		

LFG, low frequency group; HFG, high frequency group; CG, control group; IQR, interquartile range; IC 95%, 95% confidence interval for median. PFS-16, Parkinson’s Disease Fatigue Scale; kg, kilogram; cm, centimeters.

* = statistically significant improvement compared with Pre-test (*p* < 0.05); # = statistically significant worsening compared with Pre-test (*p* < 0.05).

^A^ = statistically significant differences compared with CG (p < 0.05); ^B^ = statistically significant differences compared with HFG (p < 0.05); ^C^ = statistically significant differences compared with LFG (*p* < 0.05).

### 3.3. Adverse effect

The adverse events were examined using both active monitoring (for rhabdomyolysis) and spontaneous report monitoring (for any unexpected adverse event). No side effects occurred during the testing sessions and the intervention period.

## 4. Discussion

### 4.1. Serum biomarkers

The supplementary analysis highlighted a substantial reduction in α-synuclein in serum levels of LFG that was significant over time, while no significant changes were found in the HFG and CG. This trend may reflect an improvement in the central metabolism of α-synuclein stimulated by aerobic-based intervention with low-intensity WB-EMS and indicates a general improvement in the clinical manifestations of PD patients, in the LFG. The neuroprotective role of α-synuclein in improving neuronal communication has been previously proven (Peelaerts et al., 2015), while misfolded α-synuclein aggregation is neurotoxic and correlated with PD progression.

The specific effect of aerobic-based intervention with WB-EMS on serum α-synuclein levels may reflect a general improvement in bioenergetic mechanisms, since the effects of exercise on α-synuclein are correlated with the improvement in mitochondrial dysfunction observed in PD (Koo and Cho, 2017). Conversely, after high-intensity WB-EMS training, a trend of increase of circulating α-synuclein was observed. A sort of clearance phenomenon, following a possible brain α-synuclein decrease, should be hypothesized to determine the serum α-synuclein increase.

FGF-21 plays a crucial role in suppressing abnormal α-synuclein aggregation and in neuroinflammation reduction (Fang et al., 2020). Previously, an increase in the serum FGF-21 concentration following both aerobic and strength training was assessed (Mobasseri et al., 2022). Our data on serum FGF-21 variation after treatments cannot be considered conclusive. Serum FGF-21 is not universally considered a valid biomarker for PD (Davis et al., 2020), although its possible role in regulating mitochondrial functionality in PD (Mäkelä et al., 2014). FGF-21 is a regulator of glucose and fatty acid metabolism, and its serum variation may reflect a modulation in bioenergetics metabolism after developing disease or applying therapy (Tezze et al., 2019). Our case likely involves the latter because we found increased serum FGF-21 levels in response to both types of exercise intervention, reflecting a peripheral effect of stimulations and exercise on FGF-21 release by muscle (Tezze et al., 2019), rather than a central modulation of the protein.

The CG participants showed a significant decrease in BDNF serum levels. Scalzo et al. (2010) associated PD patients’ sedentary behavior with decreased serum BDNF levels, particularly in the early stages of PD (Jiang et al., 2019). The progressive insufficient neuronal supply of BDNF and other growth factors in PD and generally in aging process determines a synaptic plasticity deficit (Palasz et al., 2020) and overexpression of α-synuclein (Kang et al., 2017). Additionally, serum BDNF has been identified as a potential biomarker for motor impairment severity (Huang et al., 2021), while physical exercise may boost serum BDNF of PD patients (Rahmani et al., 2019). Our data seem to fit well with such assumptions since we found a significant difference between the LFG and CG (ΔS). Furthermore, a positive correlation was found between motor improvement and serum BDNF increase in PD patients undergoing aerobic-based intervention with low-intensity WB-EMS, confirming that aerobic exercise increases serum BDNF levels (Wang et al., 2022). Thus, the increased serum level of this neurotrophin may reflect its increased brain availability and/or its action in promoting a relative recovery of dopaminergic neurons activity (Hyman et al., 1991).

Interestingly, we did not observe significant variation in the BDNF-related neurotrophin NGF or its precursor proNGF in patients’ sera. In particular, this latter has been recently identified as a possible biomarker for PD (Xu et al., 2018), while serum NGF was found to be decreased only in Grade I-II (according to the Hoehn and Yahr scale) PD patients (Pedre et al., 2002). Our results, although not directly contradicting the cited ones, however, seem to exclude NGF/proNGF as a possible candidate biomarker for motor improvement after physical exercise in PD patients.

### 4.2. Physical performances

The physical assessment highlighted a significant improvement in several outcomes for the LFG and HFG. Both experimental groups showed significant improvement in the arm curl test and sit-to-stand test with pre-post significant differences *vs.* the CG. Additionally, the CG worsened the sit-to-stand task during that period. An increase in lower and upper limb strength, obtained in a short time could be very promising even for PD gait and posture, counteracting the worsening observed in the CG (Cruz-Jentoft et al., 2019). Furthermore, the CG significantly worsened the 8 foot up and go test: the discontinuous pattern of this exercise (standing, walking, turning, and sitting) might have accounted for this result because it represented a substantial challenge related to the PD at the basal ganglia level (Lauzé et al., 2016). Conversely, the LFG and HFG showed improved time in the 8 foot up and go test. Because turning, getting up and sitting are more representative of daily life movements (Mollà-Casanova et al., 2022), this improvement could lead to more autonomy in the daily life management of PD patients.

Handgrip strength was not positively or negatively affected by the exercise interventions. Weaker handgrip strength in PD patients is more influenced by disease severity and development than by general strength status (Roberts et al., 2011). These finding contrasts that in studies on older people in which handgrip strength is used for assessing physical and cognitive health levels (Mcgrath et al., 2019; Shaughnessy et al., 2020).

Static and dynamic balance and walking capacity improved only in the LFG. The active movement performed by the LFG, varying the joint angles, alters the muscle length increasing the spatial recruitment (Maffiuletti, 2010). Isometric exercise, performed by the HFG, stimulates a large contraction of specific group muscles with less energy demand, preserving motor output. However, this type of training does not request accuracy in the precise amount of force control, joint-position sense, and proprioception (Lum and Barbosa, 2019). The stability and accuracy of the force production and concurrent activation of agonist and antagonist muscles, are essential requirements for walking and performance evaluated by the Tinetti test (Hortobágyi et al., 2001). Continuous rowing-ergometer exercise, in addition to determining large increases in maximal strength of the leg extensors and trunk muscles, better improved coordination (Casas et al., 2019).

### 4.3. Fatigue perception

Regarding fatigue a previous study highlighted that the exercise typology and protocol planned make patients perceive exercise as a trigger or alleviating factor for fatigue (Lin et al., 2021). Our study showed that the training-induced gain on fatigue was significantly higher in the LFG than in both the HFG and CG, and the HFG perceived significantly less fatigue than the CG. The concept that exercise counteracts the perception of fatigue is supported by the worsening of the CG over time (Thrue et al., 2022). LFG training, such as aerobic-based training, improves central fatigue in individuals with PD because of increased exercise tolerance and strength as well as coordination, that are promising means to increase the basal ganglia volume (Juvet et al., 2017). Additionally, the increased serum BDNF level, following aerobic exercise, may explain the relationship between central fatigue and LFG training. BDNF not only enhances synaptic gamma-aminobutyric acid clearance (Pierce Boyne et al., 2019) but also potentiates normal central nervous system myelination (Fletcher et al., 2018).

### 4.4. Strengths and limitations

The novel aspect of this study was the examination of the impact of WB-EMS superimposed on two exercise protocols for the first time applied on PD patients. WB-EMS superimposed on physical exercise allows a general improvement in the clinical manifestation of PD patients. However, in the present study, WB-EMS was not used to replace exercise but to supplement it and make it more effective. Consequently, it is not possible to clearly distinguish the effect of training from that of WB-EMS. Further studies may isolate the effect of the electrical stimulus produced by the device.

Although the change in different growth factors, as a result of training, may account for the change in the health and performance of PD patients, we decided to evaluate the same parameters analyzed in our previous paper in which the acute effects of a single session of WB-EMS on physical performance and serum levels of neurotrophic factors in were evaluated in PD patients (Fiorilli et al., 2021).

The data collected are not generalizable to all clinical diagnoses of PD patients because the participants were in the stage from mild (1) to moderate (3), as assessed by the Hoehn and Yahr scale. In agreement with previous data, we also noticed large variability in the changes of the studied variables, making it difficult to detect anytime significant changes.

## 5. Conclusion

These results could emphasize the strong effect of our protocols as a promising means of PD therapy. The aerobic-based intervention with low-intensity WB-EMS confirmed itself as the best choice for PD patients, improving growth factors’ release and both α-synuclein and fatigue decrease. The WB-EMS additions allowed PD patients to reduce their time of weekly exercise (20 min two times per week *vs*. one hour and a half, three times per week, such as in conventional aerobic training). Additionally, WB-EMS training was conducted in an individualized setting, a crucial component for achieving results, considering the high heterogeneity of PD patients.

## Data availability statement

The raw data supporting the conclusions of this article will be made available by the authors, without undue reservation.

## Ethics statement

The studies involving human participants were reviewed and approved by Bioethical Committee “Azienda Sanitaria Regionale Molise–ASREM” (11487/2020). The patients/participants provided their written informed consent to participate in this study.

## The WB-EMS Parkinson’s Group

Francesco Lena, Department of Medicine and Health Sciences, University of Molise, Campobasso, Italy; Nicola Modugno, Neuromed Institute IRCCS, IS, Pozzilli, Italy; Federico Quinzi, Department of Clinical and Experimental Medicine, University of Catanzaro “Magna Græcia”, Italy.

## Author contributions

AC and GF led the study design and write up. AB, MC, and AD contributed to data collection. AB, MS, and LM carried out the laboratory analyses. EI, GCas, LM, and AB led the data analysis. AC, GF, GCal, AP, AD, GG, LM, MS, and EI were involved in commenting, revising, reviewing the manuscript, and approved it. All authors had full access to the data and were responsible for the integrity of data and the accuracy of the analysis, contributing to drafting.
